# Heterogeneity of COVID-19 Risk Perception: A Socio-Mathematical Model

**DOI:** 10.3390/ijerph182111007

**Published:** 2021-10-20

**Authors:** Alfonso Gastelum-Strozzi, Claudia Infante-Castañeda, Juan Guillermo Figueroa-Perea, Ingris Peláez-Ballestas

**Affiliations:** 1 Instituto de Ciencias Aplicadas y Tecnología, Universidad Nacional Autónoma de México, Circuito Exterior S/N, Ciudad Universitaria, Mexico City 04510, Mexico; alfonso.gastelum@icat.unam.mx; 2 Instituto de Investigaciones Sociales, Universidad Nacional Autónoma de México, Mexico City 04510, Mexico; claudiainfante@prodigy.net.mx; 3 Centro de Estudios Demográficos, Urbanos y Ambientales, Colegio de México, Mexico City 14110, Mexico; jfigue@colmex.mx; 4 Rheumatology Unit, Dr. Eduardo Liceaga General Hospital of Mexico, Mexico City 06720, Mexico

**Keywords:** risk perception, heterogeneity, students, COVID-19, Mexico

## Abstract

The perception of risk has been a key element in the experiences, containment and differential impact of the COVID-19 pandemic worldwide. The complexity of this phenomenon requires the interdisciplinary integration of theoretical and methodological aspects, as this integration informs the objective of developing a mathematical proposal based on a conceptual model located within the social theory of risk at the micro-social level. The mathematical risk model used here was developed from a secondary analysis of a study of 12,649 individuals on the experiences of the COVID-19 pandemic in a population in which the quantity and quality of the information made it possible to define a risk factor and its relationship to emotions and the sources of information used. Four sequential strategies were used to construct the model: choosing the variables for the questionnaire that theoretically corresponded to the conceptual model, constructing the risk vector and initial grouping of individuals by perception of risk, modeling by using principal component analysis and applying network methods. The theoretical model of risk, proposed and constructed through the analysis of groupings by quartiles and by networks in the studied population from a social and mathematical perspective, demonstrates the heterogeneity of risk perception as manifested by differences in perception by age, gender, expression of feelings and media consulted in a university community. The knowledge and methodology generated in these analyses contribute to the body of knowledge informing the response to future epidemiological contingencies.

## 1. Introduction

The COVID-19 pandemic has produced an impact at all levels, with variable morbidity and mortality in different countries. The care and recommendations to avoid contagion and morbidity and mortality have also been different. In of the reasons that has been added to such care and following the recommendations of the health authorities has been the perception of risk that populations have [1]. In some groups, such as students, they report a low perception of risk, and in other groups, such as people with chronic diseases or in older adults, they report a high perception of risk [2].

The perception of risk has a socially and culturally determined nature [3]; an assessment of the experiences and social responses related to the perception of risk in the context of the COVID-19 pandemic in different populations is of fundamental importance, especially given the relevance of non-pharmacological measures in containing the pandemic and the varying reactions of different population groups to these measures.

This study began based on a sociologically oriented conceptual model located within the social theory of risk at the micro-social level, addressing two broad areas: risk perceptions and risk behaviors [4].

**Figure 1:**
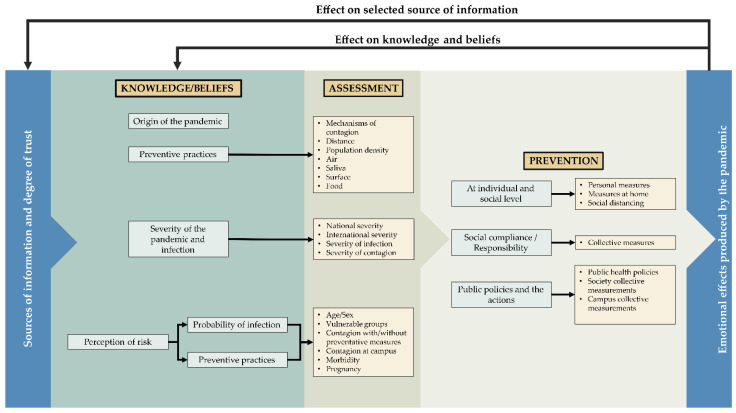
Representation of the conceptual model. The variables of age, sex, civil status (partner/no partner), level of education and institutional position within the university (student/level, academic/administrative staff) were included to characterize the population subgroups.

The conceptual model included the following topics (Figure 1):

1. Contextual variables: age, sex, civil status (partner/no partner), level of education and institutional position within the university (student/level, academic/administrative staff);
2. Knowledge and beliefs;
3. The assessment of risk perception included: perception of severity, the probability of infection and the environment;
4. Preventive practices;
5. Sources of information and degree of trust in each of them.

Therefore, the proposed objective of this study was to design a risk-perception model based on the experiences of the COVID-19 (SARS-CoV2) pandemic, building on a secondary analysis of a previous study [5] in a university population that, by virtue of the quantity and quality of the information, made it possible to define a risk factor and its relationship to emotions and the sources of information they use. Additionally, the study seeks to be a starting point for a reflection on the forms of perception and measurement of risk regarding the pandemic from a sociological perspective, coupled with a mathematical-modeling proposal.

The authors start from the hypothesis that the reflexive combination of sociology with a mathematical proposal will allow for an identification of the perception of risk that accounts for the diversity of experience of the subjects in their different dimensions; this is where the age, gender and behaviors of the subjects in the face of the pandemic allow for an approach to the complexity of the experience of the pandemic in this population. To the best of our knowledge, this is the first analysis to use this approach to risk perception and its associated factors in the early stages of the pandemic in a university community in Mexico, and it is intended to complement similar studies in other countries [6,7,8,9]

## 2. Materials and Methods

A secondary analysis was conducted on a survey administered online in Mexico City from 6 April to 26 May 2020. Individuals aged 18 and over from the university community were invited to participate and voluntarily agreed to respond to the survey published on the official university website [10].

We used the validated questionnaire OUSOCIAL-COVID-19 [10], which is divided into eight dimensions: (1) sociodemographic characteristics of the population, (2) knowledge of the pandemic; (3) means of information; (4) perception of risk and severity of the pandemic; (5) effects of the pandemic on mental health; (6) effects of the pandemic at the personal and family level; (7) current state of health, COVID-19 disease in individuals, their social network and comorbidities; (8) communication and relations with the university community; and (9) opinions on the measures adopted to mitigate the pandemic (Supplementary Material Table S1). Previously published reports may be consulted for details on the primary study [5,10].

Descriptive analysis: A descriptive analysis was performed on the demographics of the respondents who agreed to participate and completed the entire questionnaire. Central tendency and dispersion measures were described for continuous variables and measurements of absolute and relative frequency for categorical variables. The normality of the variables was corroborated with the Shapiro–Wilk test. Inferential statistics tests were conducted to determine differences between the groups with different risk perceptions. These tests included chi-square tests for nominal sociodemographic variables.

### 2.1. Construction of the Model

#### 2.1.1. Risk Model

Four sequential strategies were used to construct the risk model: (1) selection of the variables by the dimensions of the questionnaire that theoretically corresponded to the proposed theoretical model (see Introduction and Supplementary Material Table S1), (2) construction of the risk vector and initial grouping of individuals by perception of risk, (3) modeling by using categorical principal component analysis (CAT-PCA) and (4) applying network methods.

#### 2.1.2. Definition of the Theoretical Model

The population’s risk perception with respect to the COVID-19 pandemic was calculated by using questions organized in the three dimensions of risk from the conceptual model: knowledge, perception/severity of risk assessment, and description and evaluation of preventive measures ( Supplementary Material Table S1). Each of the questions was converted from its categorical representation to a series of dummy variables, where the sign was allocated based on how the responses line up with the precautions and knowledge expected during the COVID-19 pandemic according to the scientific knowledge at that time.

#### 2.1.3. Construction of the Risk Vector and Initial Grouping

The dichotomous representation with the sign of the variables was used to define the individual vector, defined by the sequence of responses obtained. Said vector was used to calculate the value of risk perception (sum of all elements of the vector). The risk value (risk perception) was used to provide an initial grouping to the subjects in quartiles. Use of this strategy of division by quartiles prevents a minimum risk value from being defined and the population is divided into similar percentages in each quartile, from the first quartile that contains the individuals with the lowest perception of risk to the fourth that groups the individuals with the highest risk perception. In this way we sought to obtain the relation across quartiles, demographic data, emotions and sources of information reported by each individual and thus describe the range of perceptions generated by the pandemic in the participating population.

#### 2.1.4. Information Modeling

Finally, the risk vector was used to assess the similarities among the subjects based on their responses. For this, a similarity value among the subjects was calculated by using the cosine similarity method [11]. Cosine similarity is a measure of how similar two vectors are, it measures the similarity in orientation without considering the magnitude. Due to the high number of variables comprising the risk vector and the significant variations among the subjects, the dimensionality of the risk vector was reduced by using the CAT-PCA method, using the function “princals” of the Gifi R package [12] based on the work of Albert Gifi [13] before calculating the similarity value. This method allows for a description of the population through the new components constituted by the variables that contribute to a greater extent to the variance in the population. From the original 166 variables, 80 principal components were selected by using a cutoff that keep the principal components that describe the 80 percent of the variance [14].

The selected principal components define a vector per individual that was used to perform the similarity calculation. The obtained similarity values range from −1 to 1. Negative values indicate dissimilarity; those with values of 0.5 (50% similarity) were used as the cutoff point. Subsequently, all pairs of individuals with a similarity value greater than cutoff point are set to be related. These relations () edges were used to perform the network analysis [15] of the individuals in order to model the interaction among subjects based on their perception of risk.

#### 2.1.5. Network Method

To construct the network model, individuals are represented as nodes, and their interrelations with other nodes are defined by using connection axes. The axes are obtained by using the cosine similarity between each pair of individuals. If the similarity value obtained is greater than 0.5, a new axis is defined between the pair of subjects.

The list of nodes, along with the list of node axes, are used to define the model to be solved by networking techniques. The network was simulated using Gephi software [16] with the ForceAtlas2 algorithm [17]. The distribution of nodes obtained by the network method was grouped by using the DBSCAN (density-based spatial clustering) algorithm [18]; the groups obtained define the individuals (nodes) with a greater degree of similarity between them.

This project was approved by the Ethics, Research and Biosafety Committees of the Hospital General de Mexico “Dr Eduardo Liceaga” (DI/20/301/03/22) and the university authorities of the Universidad Nacional Autonoma de México, (UNAM); the recommendations of the Pan American Health Organization for conducting Public Health research during the COVID-19 pandemic were also followed [19].

## 3. Results

A total of 12,649 individuals from a university community with an average age of 33.5 years participated; of these, 7841 (62.0%) were women. The distribution of risk perception by quartiles (QR1–QR4) in relation to gender and age may be observed in Table 1.

**Figure 2:**
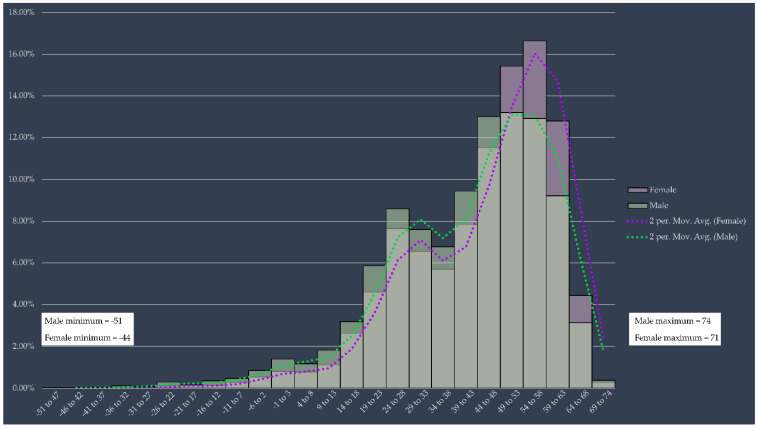
Numerical distribution of the perception of risk in the population.

In Figure 2, a histogram is presented with the bimodal distribution of the risk values obtained in the population. The bimodal distribution shows an initial distribution of the individuals with respect to the risk perception; the lower the risk value obtained, the lower the perception of risk. The first mode shows the subset of individuals that have a low-risk perception; the second mode shows the individuals that perceive the highest risk. Risk-perception values ranged from −51 to 74, with a significant difference observed between males (male QR (MQR) = −51 to 74) and females (female QR (FQR) = −44 to 71).

The emotional reactions experienced in order of frequency in the population were alertness (8188, 64.73%), worry (7819, 61.82%), isolation (6486, 51.28%), anxiety (5904, 46.68%), alarm (4523, 35.76%), boredom (4227, 33.42%), confusion (3885, 30.71%), fear (3721, 29.42%) and depression (3130, 24.75%).

**Figure 3:**
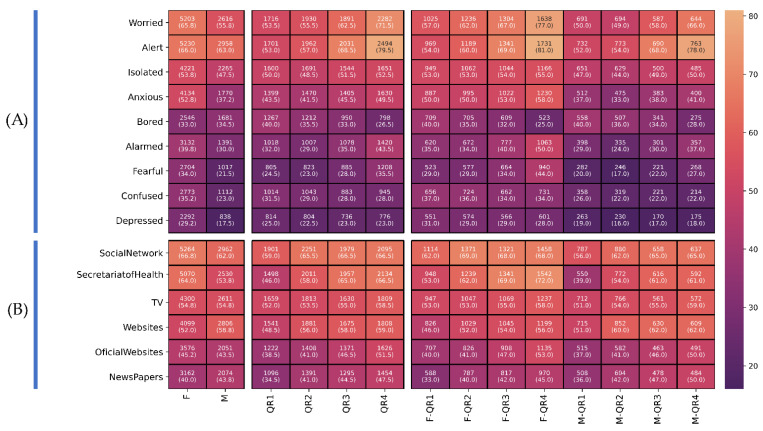
Relation between the perception of risk and gender and the emotions. (**A**) reported by the individuals and the media consulted (**B**). All groups are reported with a *p* < 0.01 illustrating the total number of individuals (percentage of the total group). The more intense the colour, the higher the frequency on the scale.

The different groups formed by the quartile of risk perception, emotions and gender are illustrated in Figure 3A. The intensity of the red color indicates the frequency of individuals experiencing each emotion compared to the total number of individuals in each group. Variables were put in order according to the similarity of behavior and frequency in the population.

The emotions of alertness and worry behave in a similar manner in both genders, escalating as the perception of risk increases (alertness (QR1: 53% and QR4: 79.5%) and worry (QR1: 53.5% and QR4: 71.5%)). A significant difference related to gender was observed in the values for isolation (53.8% in females and 47.5% in males) and anxiety (52.8% in females and 37.2% in males). In both populations it is observed that the greater the perception of risk, the greater the presence of both emotions, this pattern being greater in females. A pattern was also observed in relation to boredom wherein the greater the perception of risk, the lower the level of boredom (QR1: 37% and QR4: 34%), and this pattern is shared by both genders. Significant differences by gender and perception of risk were observed in the emotions of alarm and fear. These emotions are found to an even lesser extent in males with a low perception of risk (alarm (MQR1: 29% and FQR4: 37%) and fear (MQR1: 20% and MQR4: 27%)). Confusion and depression occur in a similar distribution across quartiles of risk and gender, occurring to a greater extent in females with lower perception of risk [confusion (FQR1: 37% and FQR4: 34%) and depression (FQR1: 31% and FQR4: 28%)).

The media most consulted were social media (8226, 65.03%), announcements by the Ministry of Health (7600, 60.08%), television (6911, 54.64%), websites (6905, 54.59%), official websites (5627, 44.4%) and newspapers (5236, 41.3%). The results of media use by gender and risk perception are illustrated in Figure 3B.

Social media and reports from the Ministry of Health were more consulted by females (social media (F: 66.8% and M: 62%) and Ministry of Health reports (F: 64% and M: 53.8%)). In both genders the greater the perception of risk, the greater the number of individuals consulting them (social media (QR1: 59% and QR4: 66.5%) and Ministry of Health reports (QR1: 46% and QR4: 66.5%)).

The use of television was similar in both genders, with increased use corresponding to a greater perception of risk (QR1: 52% and QR4: 58.5%). Websites and newspapers were the media consulted more by males (websites (F: 52% and M: 58.8%) and newspapers (F: 40%, M: 43.8%)); consultation of these media increases with respect to the perception of risk in both genders (websites (QR1: 48.5% and QR4: 59%) and newspapers (QR1: 34.5% and QR4: 47.5%)). Official websites are more consulted as the perception of risk increases (QR1: 38.5%, QR4: 51.5%).

**Figure 4:**
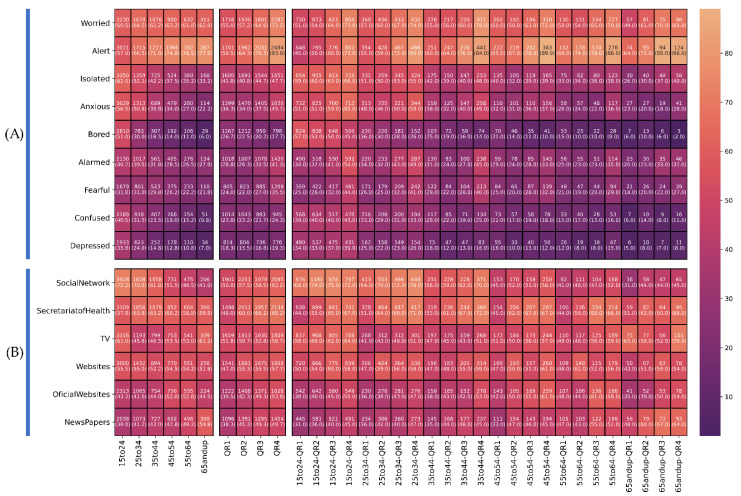
Relation between the perception of risk and age and the emotions. (**A**) reported by the individuals and the media consulted (**B**). All groups are reported with a *p* < 0.01 illustrating the total number of individuals (percentage of the total group). The more intense the colour, the higher the frequency on the scale.

The relations between risk perception, age and emotions reported by respondents are illustrated in Figure 4A. Worry and alertness have similar values in most age groups, except for the feeling of alertness in the 15-to-24 age group, where the frequency is lower (57.0%), and it is higher in the 65-years-and-over group (77.5%). In contrast, for the emotions of anxiety, isolation, fear, alarm, depression, confusion and boredom, there is a decrease in the number of individuals who report these as age increases (anxiety (15 to 24: 56.5%, and 65 and over: 22.2%), isolation (15 to 24: 62.0%, and 65 and over: 33.2%), fear (15 to 24: 31.8%, and 65 and over: 21.8%) and alarm (15 to 24: 40.2%, and 65 and over: 27.0%)) and increasing according to the perception of risk (anxiety (QR1: 36.3% and QR4: 45.7%), isolation (QR1: 41.8% and QR4: 47.7%), fear (QR1: 24% and QR4: 35.5%) and alarm (QR1: 28.8% and QR4: 41.3%)). Depression, confusion and boredom decrease as age increases (depression (15 to 24: 35.8%, and 65 and over: 7.0%), confusion (15 to 24: 40.5%, and 65 and over: 9.8%) and boredom (15 to 24: 51%, and 65 and over: 6.0%)), where there is a similar behavior of depression for different quartiles of risk perception, and boredom decreases as the perception of risk increases (QR1: 26.7% and QR2: 17.7%).

The relations between risk perception, age and the means of communication consulted are illustrated in Figure 4b. Only two means of communication have a clear relationship with respect to age groups: social media, in which use decreases as age increases (15 to 24: 72.2%, and 65 and over: 41.0%) and consulting newspapers, that increases with age (15 to 24: 38.0%, and 65 and over: 59.8%). In all cases an increase in the consumption of the media as the perception of risk increases may be observed.

**Figure 5:**
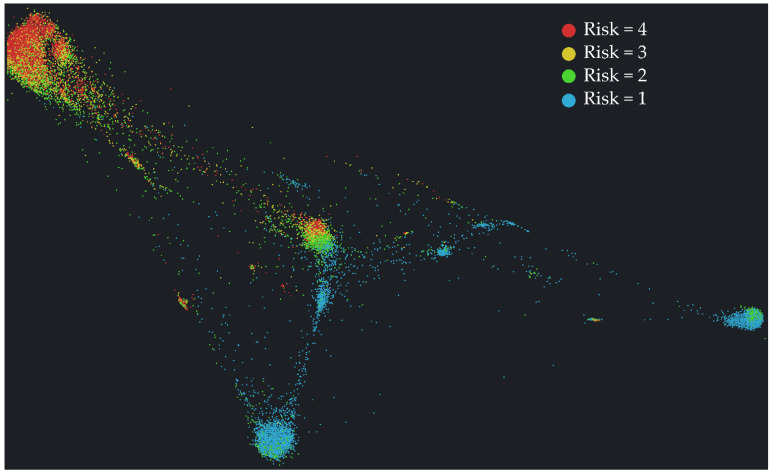
Network obtained illustrating the distribution of individuals based on the similarity values among them.

In Figure 5, a graphic representation of the network resulting from the modeling of attraction and repulsion of individuals (each represented by a circle) is illustrated, where the final position of each individual within the chart depends on the attraction to other similar individuals and repulsion toward different individuals with respect to the risk vector (vector defined by the dummy representation of the variables used for the calculation of the perception of risk; see Supplementary Material Table S1).

To assist in the visual identification of groups formed by individuals with similar risk vectors, the color of each individual is given by the quartile of risk perception to which he or she belongs. The size of the different groups observed depends on the number of subjects with a similar behavior. As may be observed, a group formed by most of the subjects with a high perception of risk is found at the top of the graph, because their behaviors with respect to the pandemic are homogeneous insofar as they follow the indications for care and their opinions coincide with the recommendations of the healthcare authorities. In contrast, individuals with a lower perception of risk are grouped in different locations on the graph (blue circles), because the reasons why they do not perceive risk are highly heterogeneous.

**Figure 6:**
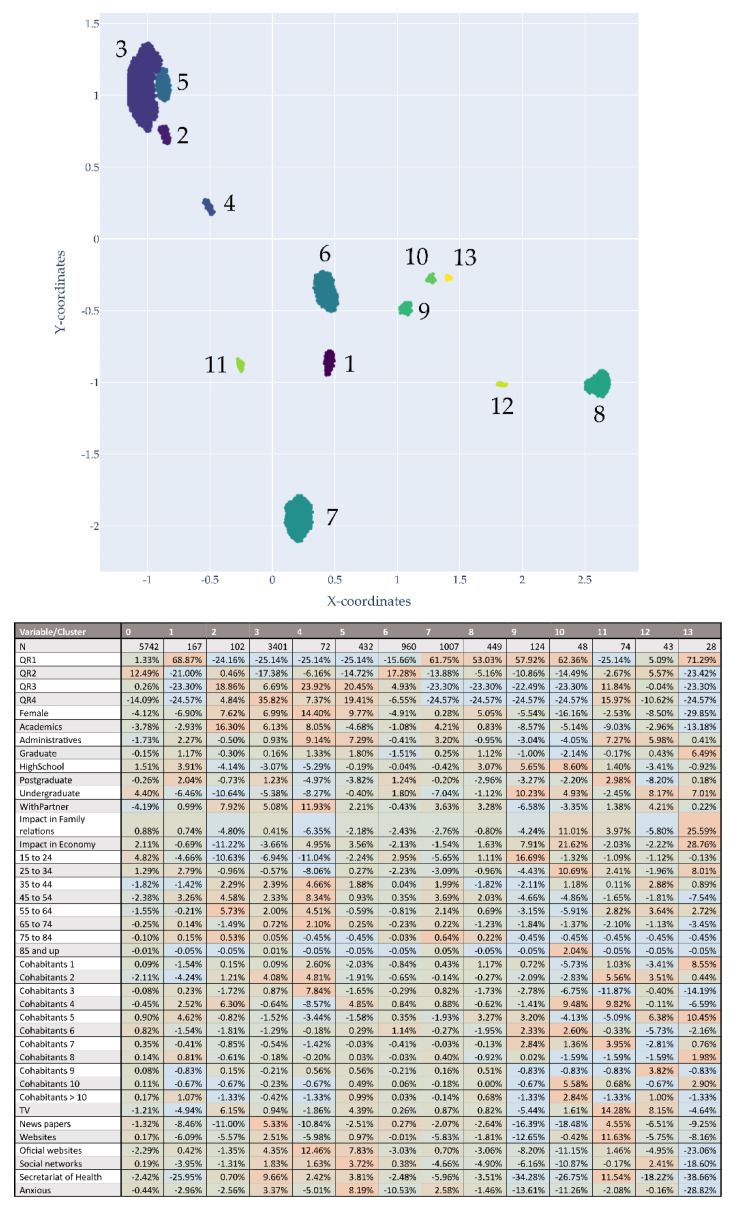
Groups obtained after the network and cluster methods. The table below illustrates the new population distribution by group, with prevalence of risk perception, gender, age, emotions and media consumption by group. The colour red indicates a higher prevalence compared to the group resulting from the network.

The top of Figure 6 provides a graphic representation of the groups obtained by the clustering method, followed by a table with the values of age, gender, risk perception, emotions and means of communication of each of these clusters. The 13 groups with the highest number of individuals are presented. The smallest group contained 28 individuals and the largest 3401. These groups represent 54.6% of the population (*n* = 6907); the rest have a greater dispersion to the area of grouping or belong to groups with numbers too small for interpretation.

As may be observed in the chart, Cluster 3 has the largest number of respondents and consists primarily of individuals with a high perception of risk, a larger number of females at 68.9% (3.57% higher than the general population) and a lower percentage of young people aged 15 to 24 (2% less). As may be observed, all the emotions are represented to a greater extent in this group, with the exception of boredom and confusion (by a very low percentage). In addition, this group consumes all media to a greater extent than the general population with the exception of television by only 0.07%. Except for age, Cluster 5 has a similar profile with a high proportion of respondents with a high perception of risk, with a greater number of women, but in this case with higher proportions of individuals under 44 years of age. With a greater representation of younger subjects, it is not surprising to find that, in this cluster, they report all emotions, including boredom and confusion; this finding also distinguishes this group from Cluster 3.

Cluster 6, in which a medium perception of risk is found, mostly comprises males under 24 years of age reporting boredom and consuming social media and websites as their primary means of information.

Clusters 7 and 8 have a high proportion of subjects with a lower perception of risk, but in Cluster 7, an increased number of males is observed (3.15%), and in Cluster 8, an increase in females (1.63%). With respect to ages, Cluster 7 has fewer subjects under 34 years of age and Cluster 8 fewer subjects under the age 55. The subjects in Cluster 7 reported having experienced more emotions than those of the general population and those in Cluster 8 reported higher levels only in the emotions of isolation, boredom, confusion and depression. Both groups consume less media than the general population.

## 4. Discussion

The theoretical model of risk proposed and constructed through the analysis of groupings by quartiles and by networks in the studied population, from a social (knowledge, perception and severity of the risk, as well as the opinion and compliance with the preventive measures adopted) and mathematical perspective, demonstrates the heterogeneity of risk perception. This heterogeneity is manifested by the differences in perception by age, gender, expression of feelings and media consulted in a university community.

The understanding of the complex phenomenon of risk perception in a pandemic with the severity and impact of COVID-19 is significantly enhanced with the use of different conceptual models—disciplinary, theoretical and methodological—whose integration allows for a better understanding of the problem of the risk perception of pandemics as socially situated phenomena [20], culturally determined and experienced differentially.

Populations with a low perception of risk are in a position of greater vulnerability. In addition, the pandemic has revealed, reinforced and created new vulnerabilities and is having secondary impacts that are being increasingly identified the future project of millions of university students around the world was altered during the pandemic. Thousands of university students abandoned their life projects for good; young female students, in particular, reported perceiving more risks and more negative social and psychological effects [21].

The experience of the pandemic has revealed other vulnerabilities, such as the circumstance of being male and therefore, perceiving the risk and the need to take care of oneself to a lesser extent, as shown in the results analyzed here. These results coincide with that which [22] has reported in monitoring the perception of risk in a British population. Different results of the male responses obtained in this and other analyses [5], seem to indicate a perception of invulnerability to the risks of the pandemic by certain groups of men. A broader explanation of the results observed among the male participants may be obtained through other studies conducted in different cultural contexts, as the study of men’s health and disease processes has established categories to account for the peculiarity of the results observed in this group, described elsewhere as “suicidal neglect” [23], ”masculinity as a risk factor” [24,25], “dying like a man” [26,27], “being a man is bad for your health” [28,29,30], or “omission of self-care” [31,32] and, in the end, “the fragility of the invulnerable” [33], due to the fact that men tend to underestimate the perception of the risks they regularly face.

It is important to identify the vulnerability of subpopulations and develop specific strategies to mitigate the effects of such vulnerability. Mathematical models built based on conceptual models can anticipate consequences and contribute to this purpose [34,35].

The results obtained with the mathematical model allow us to identify how individuals are grouped depending on whether they have similar levels of perception of risk, based on the homogeneity or heterogeneity in the factors that characterize that perception of risk. This heterogeneity identified in the levels of perceived risk helps us understand that even within a single population with similarities such as that of the university community, there are individual differences that occur due to the cultural context, which were indirectly identified with the theoretical mathematical model. In addition, this model allows us to reflect on the fact that heterogeneity exists even in an academic community, with a similar availability of scientific and epidemiological sources of information.

On the other hand, in terms of the possibility of modeling these behaviors and being able to predict how the pandemic will unfold, new problems arise to be considered according to the results obtained from using network and grouping methods that indicate that behaviors related to risk are heterogeneous. When analyzing the behaviors of individuals, it is important to ask certain questions: For example, is our system ergodic or non-ergodic? Do people act with an irrational fear or are their expectations of the impact of COVID-19 justified? Moreover, which group is at the greatest social risk considering the events measured?

Prior to the outbreak of the COVID-19 pandemic in the Americas, a hypothesis on the variability of personal responses to catastrophic events had posed the question as to whether such variability was the result of a cognitive bias due to the “probability neglect” of society to assess fear, since some experts consider this fear irrational [36,37]. Clearly the fact that individuals misjudge the risk of a given phenomenon and make decisions accordingly can have an impact on society, even if the risk is unlikely. The slow response to a pandemic [38,39], moreover, may create more problems when trying to contain it. This is the dilemma between assessing a risk or an uncertainty, since the former is located in an assumption of ergodicity and the latter is in a non-ergodic world.

The impact and opinions of a group of individuals from a university community following the outbreak of the COVID-19 pandemic allowed us to evaluate the usefulness of applying the concepts of “probability neglect” in the initial reactions of fear of contagion or disease. The variability of reactions of fear may be due to an exponential-growth bias, that is, the tendency to linearize exponential functions when assessing them intuitively, dismissing the risk of exponential growth of the pandemic [40].

In this study we found a diversity of responses based on actions and opinions that subjects in the same community have in the face of the COVID-19 pandemic. Given these observations, the following question arises: can we model the response to the risk of infection by SARS-CoV-2 or becoming ill with COVID-19 as an ergodic system, as proposed in the areas of economics [41], where within the model capabilities predictions of the pandemic can be made considering previous social behaviors, or as a non-ergodic system [40]? Where the complexity of the behavior points to changes on the system that cannot be easily predicted. This poses a complex question: what predictions can be made from the data analyzed? A potential response would be that, based on an ergodic model, we can predict a low-risk perception on young male population, or we can instead use the uncertainty and heterogeneity of the responses to define the problem as a non-ergodic system. The latter model would allow us to consider the complexity of a heterogeneous community with heterogeneous responses to a pandemic, where new uncertainties at different stages of the pandemic must be consider. However, the variations found among populations with low perceptions of risk in this study may indicate that we will always have a level of uncertainty that requires us to maintain the observation and monitoring of communities to have a better action plan at every juncture of this and future pandemics, and with this we will have new serious and complex problems in the management of resources and institutions in a non-ergodic post-pandemic world [42], where our models need constant data input to stay relevant in the description of the society with respect to the pandemic.

This study has several limitations, from the cross-sectional design where causal associations cannot be made, being administered online, which produces a potential participation bias due to accessibility to technology; memory bias and the fact that the population studied is not a representative sample and is restricted to the university community (students, academic and administrative staff) [43].

The strengths of this study are that it was able to draw from a large sample, with an analysis based on a conceptual model with a sociological approach from which a mathematical theoretical model of risk perception was constructed and tested.

## 5. Conclusions

In this article, we sought to analyze the perception of the risk of COVID-19 by a university community through an online instrument addressing eight dimensions to allow for a sociodemographic analysis, while identifying perceptions of possible effects of the pandemic, sources of communication about it and opinions on the measures adopted to combat it and ensure self-care.

Here we sought to further characterize variants of risk perceptions in terms of specific characteristics of the subjects to build a mathematical model that allows for the identification of multifactorial associations with respect to said perception of risk and, in consequence, the definition of strategies to address it. These strategies combine information, assertiveness and cultural learning (by age and gender) to identify clusters; however, multidisciplinary analyses are required to promote actions that can enhance the preparedness and responses of public actions for possible future pandemics. Such strategies cannot be seen in a linear and reductionist manner, since the analyses developed illustrate the multiple dimensions that come into play when identifying certain patterns in the actions, perceptions, experiences and opinions of the university population studied.

The models developed, together with the analysis of networks and clusters, reflect the heterogeneity of emotional responses, as well as the type of information consulted in the multidimensional perception of risk, which leads us to propose that risk models should capture the heterogeneity and the non-ergodicity of the behaviors of populations in order to implement strategies that account for such heterogeneity.

The combination of a sociological approach and mathematical modeling offers good prospects for going beyond simplistic explanations by bringing into focus the complexity of the phenomena under study.

## Figures and Tables

**Table 1:** Population distribution by quartiles of risk, gender and age.

**Gender/Risk** **Quartile**	**1** ***n* (%)**	**2** ***n* (%)**	**3** ***n* (%)**	**4** ***n* (%)**	**Total** ***n* (%)**	*p* **
Male	1395 (11.0)	1422 (11.0)	1015 (8.0)	976 (8.0)	4808 (38.0)	*p* < 0.01
Female	1785 (14.0)	1992 (16.0)	1932 (15.0)	2132 (17.0)	7841 (62.0)	
Total	3180 (25.0)	3414 (27.0)	2947 (23.0)	3108 (25.0)	12649 (100.0)	
**Age/Risk Quartile**	**1** ***n* (%)**	**2** ***n* (%)**	**3** ***n* (%)**	**4** ***n* (%)**	**Total** ***n* (%)**	*p* **
15 to 24	1438 (11.0)	1603 (13.0)	1296 (10.0)	1100 (9.0)	5437 (43.0)	*p* < 0.01
25 to 34	646 (5.0)	723 (6.0)	647 (5.0)	585 (5.0)	2601 (21.0)	
35 to 44	416 (3.0)	385 (3.0)	366 (3.0)	528 (4.0)	1695 (13.0)	
45 to 54	339 (3.0)	329 (3.0)	309 (2.0)	428 (3.0)	1405 (11.0)	
55 to 64	225 (2.0)	242 (2.0)	222 (2.0)	322 (3.0)	1011 (8.0)	
65 to 74	96 (1.0)	113 (1.0)	96 (1.0)	132 (1.0)	437 (3.0)	
75 to 84	17 (0.0)	19 (0.0)	9 (0.0)	12 (0.0)	57 (0.0)	
85 and over	3 (0.0)	0 (0.0)	2 (0.0)	1 (0.0)	6 (0.0)	
Total	3180 (25.0)	3414 (27.0)	2947 (23.0)	3108 (25.0)	12649 (100.0)	

Risk quartile value of 1 indicates the lowest perception of risk, 4 the highest. ** Chi-square with significance of *p* < 0.01.

## Data Availability

The data that support the findings of this study are openly available in Open Science Framework at http://doi.org/10.17605/OSF.IO/UPJDE, HETEROGENEITY OF COVID-19 RISK PERCEPTION: A SOCIO-MATHEMATICAL MODEL (Accessed on 18 October 2021).

## Supplementary Materials

The following are available online at https://www.mdpi.com/article/10.3390/ijerph182111007/s1, Table S1: List of questions used for the calculation of the risk perception vector and value, organized into the three risk dimensions of the conceptual model: Knowledge, perception/severity of risk assessment, and description and evaluation of preventive measures.
