# Supplementary material for: Heterogeneity of COVID-19 Risk Perception: A Socio-Mathematical Model
Source: Int J Environ Res Public Health. 2021 Oct 20;18(21):11007. doi: 10.3390/ijerph182111007 (PMC8582984; doi:10.3390/ijerph182111007)
Supplement: Supplementary file 1 [file ijerph-18-11007-s001.zip › ijerph-1383470-supplementary.pdf]

Table S1. List of questions used for the calculation of the risk perception vector and value, organized into the three risk dimensions of the conceptual model: Knowledge, perception/severity of risk assessment, and description and evaluation of preventive measures.

			**Knowledge**		
Q10	You believe that the origin of the coronavirus (COVID−19) epidemic is due to:		Q11	Which populations are most at risk if you contract the COVID−19 coronavirus?	
	Climate change	1		Children	−1
	Biological changes	1		Young people	−1
	Social changes	1		Adults under 60 years of age	−1
	Genetic manipulation	1			
	Lack of hygiene	1		Older adults (60 years and older)	1
	Poverty	1			
	Human interventions in nature	1			
	National/international political manipulations	1		People with chronic diseases (diabetes, hypertension, cancer, obesity among others)	1
	Bioterrorism/terrorism	1			
	An invention of the authorities	−1		Pregnant	1
	Economic crisis	1		Other populations	−1
	Not known	−1		All equally	−1
Q12	Do you know through what mechanisms a person can be infected by the COVID−19 coronavirus?		Q13	How much do you think you know about coronavirus (COVID−19) infection?	
	Physical closeness to a person without symptoms	1		A lot	1
	Physical closeness to a person with symptoms	1		Pretty much	1
	Closeness to someone who recently returned from abroad	1			
	Through sneezing or drops of saliva	1		Regular	−1
	Touching surfaces (furniture, doorknobs, doors, floor, objects) contaminated with the virus	1		Little	−1
	In the food	−1			
	In the air	−1		Nothing	−1
	Where there are many people	1			
			**Perception/severity of risk**		
Q17	How would you rate the situation of the coronavirus (COVID−19) epidemic in Mexico?		Q18	How would you describe the situation of the coronavirus (COVID−19) epidemic in the world?	
	Very serious	1		Very serious	1
	Grave	1		Grave	1
	Regular	−1		Regular	−1
	Little serious	−1		Little serious	−1
	Nothing serious	−1		Nothing serious	−1
Q20	How severe do you consider coronavirus (COVID−19) infection to be?		Q21	How likely do you think it is that you will catch the coronavirus (COVID−19)?	
	Very serious	1		Highly probable	1

	Grave	1		Very likely	1
	Regular	−1		Regular	−1
	Little serious	−1		Little	−1
	Nothing serious	−1		Nothing	−1
P43 (1)	If you don't take preventive measures against coronavirus (COVID−19): How likely are you to get infected?		P43 (2)	If you don't take preventive measures against coronavirus (COVID−19): How likely is it that someone in your family or at home will be infected?	
	Very high risk	1		Very high risk	1
	High risk	1		High risk	1
	Neither high nor low risk	−1		Neither high nor low risk	−1
	Low risk	−1		Low risk	−1
	Very low risk	−1		Very low risk	−1
P48	How much do you think the following collective actions taken by the authorities to confront the coronavirus (COVID−19) epidemic serve?		P50	Do you think that the risk of contagion when returning to classes and work activities at UNAM is?:	
	Suspension of classes				
	Cancellation of cultural, sporting and artistic events			Very high	1
	Closure of cinemas, museums, theatres, bars				
	Closure of parks and zoos			High	1
	Hygienic prevention measures requested from society				
	Special operation to monitor hygiene in transport			Moderate	−1
	Cancellation of activities in religious spaces (temples, churches)				
	Temporary suspension of activities in commercial establishments			Low	−1
	Suspension of non-essential economic activities				
	Apply to stay at home			None	−1
	Much	1			
	A lot	1			
	Regular	−1		I do not know	−1
	Little	−1			
	Nothing	−1			
**Preventive measures**					
P19	The preventive measures that have been taken in the country due to the coronavirus (COVID−19) epidemic seem to you:		P42	Of the following measures to avoid contagion by coronavirus COVID−19 mention if you have done them and if you consider them useful:	
				Use of face masks or face masks	1
	Very excessive	1		Frequent hand washing	1
				Use of disinfectant gel	1
				Disinfect the house	1
	Excessive	1		Do not shake hands or kiss	1
				Avoid touching your face (eyes)	1

	Regular	−1
	Enough	−1
	Insufficient	−1
P48	How much do you think the following collective actions taken by the authorities to confront the coronavirus (COVID−19) epidemic serve?	
	Suspension of classes	
	Cancellation of cultural, sporting and artistic events	
	Closure of cinemas, museums, theatres, bars	
	Closure of parks and zoos	
	Hygienic prevention measures requested from society	
	Special operation to monitor hygiene in transport	
	Cancellation of activities in religious spaces (temples, churches)	
	Temporary suspension of activities in commercial establishments	
	Suspension of non-essential economic activities	
	Apply to stay at home	
	Much	1
	A lot	1
	Regular	−1
	Little	−1
	Nothing	−1

	Handrail washing	1
	Do not wear street shoes at home	1
	Coughing or sneezing at the inner angle of the arm	1
	Change and wash the clothes you used on the street	1
	Avoid using public transport	1
	Avoid going to closed or open public places	1
	Avoid meeting with groups of many people	1
	Avoid going to school or work	1
	Keep a distance of one and a half meters from the people you interact with	1
	Do not leave your house	1
	Do not travel	1
P52	Point out whether you agree or disagree with the following statements:	
	Returning to work does not mean eliminating all preventive measures	1
	Preventive measures must continue to be part of our daily lives for some time	1
	It is necessary to follow the preventive measures proposed by the UNAM authorities when we return to activities	1
	The main actions to reduce risks "are in our hands"	1
	We are all co-responsible for risk prevention	1
	One of the fundamental values of the university community is solidarity among its members	1
	Solidarity with preventive measures among the UNAM community can be a very positive example for all Mexicans.	1
	It is everyone's responsibility to make up for lost time in our UNAM activities	1
